# Dry immersion rapidly disturbs iron metabolism in men and women: results from the VIVALDI studies

**DOI:** 10.1038/s41526-024-00399-z

**Published:** 2024-06-15

**Authors:** Mathieu Horeau, Nastassia Navasiolava, Angelique Van Ombergen, Marc-Antoine Custaud, Adrien Robin, Martine Ropert, Inês Antunes, Marie-Pierre Bareille, Rebecca Billette De Villemeur, Guillemette Gauquelin-Koch, Frédéric Derbré, Olivier Loréal

**Affiliations:** 1 INSERM, University of Rennes, INRAE, UMR 1241, AEM2 Platform, Nutrition Metabolisms and Cancer (NuMeCan) institute, Rennes, France; 2 Laboratory “Movement Sport and Health Sciences” EA7470, University of Rennes/ENS Rennes, Rennes, France; 3 Univ Angers, CRC, CHU Angers, Inserm, CNRS, MITOVASC, Equipe CARME, SFR ICAT, Angers, France; 4 European Space Agency (ESA), Noordwijk, The Netherlands; 5 Department of Aerospace Engineering, Texas A&M University, College Station, TX USA; 6 Telespazio Belgium S.R.L. for the European Space Agency, Noordwijk, The Netherlands; 7 Institute of Space Physiology and Medicine (MEDES), Toulouse, France; 8 Centre National d’Etudes Spatiales, Paris, France

**Keywords:** Physiology, Adaptive clinical trial

## Abstract

Iron is essential for cell respiration, muscle metabolism, and oxygen transport. Recent research has shown that simulated microgravity rapidly affects iron metabolism in men. However, its impact on women remains unclear. This study aims to compare iron metabolism alterations in both sexes exposed to 5 days of dry immersion. Our findings demonstrate that women, similarly to men, experience increased systemic iron availability and elevated serum hepcidin levels, indicative of iron misdistribution after short-term exposure to simulated microgravity.

## Introduction

Exposure to real or simulated microgravity induces skeletal muscle wasting, osteoporosis, and anemia^1–3^. Alterations in iron metabolism could contribute to these imbalances due to the central role of iron in energy metabolism, cell respiration, oxygen transport, and muscle function^4^. Previous research has demonstrated that young men exposed to real or simulated microgravity rapidly exhibit iron redistribution, characterized by increased serum iron availability (i.e., serum iron and transferrin saturation levels) and spleen iron sequestration^5,6^. This redistribution is accompanied in both human and rodent males by an increase of both hepcidin mRNA levels in the liver and peptide levels in plasma^5,7–9^. Hepcidin, which limits the membrane expression and activity of ferroportin, the only known exporter of Fe^2+^ from cells, especially from spleen macrophages and enterocytes^10^—could potentially contribute to the early iron redistribution observed in male astronauts and bedridden patients.

Despite the increasing number of female astronauts and the known sex-related differences in basal iron metabolism regulation^11,12^, all current data have been collected only from male subjects. To address this gap, we recently participated in the AGBRESA clinical study, which involved 16 male and 7 female participants exposed to 60 days of head-down tilt bed rest^13^. In this group of participants, our preliminary findings suggested that women exhibit increased iron availability in plasma after 6 days of bed rest. However, unlike their male counterparts, they did not show a concomitant increase in serum hepcidin levels^13^. Furthermore, after 60 days, female subjects no longer exhibited an increase in transferrin saturation level, unlike male participants, suggesting sex-specific differences in the regulation of iron metabolism over the long term^13^. This excess of plasma iron could progressively lead to oxidative stress in organs and raise the possibility of exacerbating organ damage in astronauts, particularly osteoporosis, muscle atrophy, cancer, or liver injuries. Additionally, a better understanding of the effects of microgravity on iron metabolism could also enhance the monitoring and treatment of bedridden patients on Earth, who experience a similar degree of physical inactivity. To further substantiate these short-term results, we investigated the regulation of iron metabolism in both sexes exposed to simulated microgravity. The first campaign included eighteen female participants (VIVALDI) and the second nineteen male participants (VIVALDI 2) exposed to 5 days of dry immersion, a ground-based model mimicking microgravity^14^. Both well-controlled campaigns were conducted at MEDES (Toulouse) and similarly set up (i.e., diet, biological sampling, and management).

Serum ferritin, an iron-storage protein, is widely recognized as a marker of body iron stores in the absence of inflammation. Serum transferrin, the iron transporter, becomes increasingly saturated as circulating iron levels rise^10^. The transferrin saturation coefficient is calculated as the ratio of iron to transferrin and serves as a marker of plasma iron availability.

**Fig. 1:**
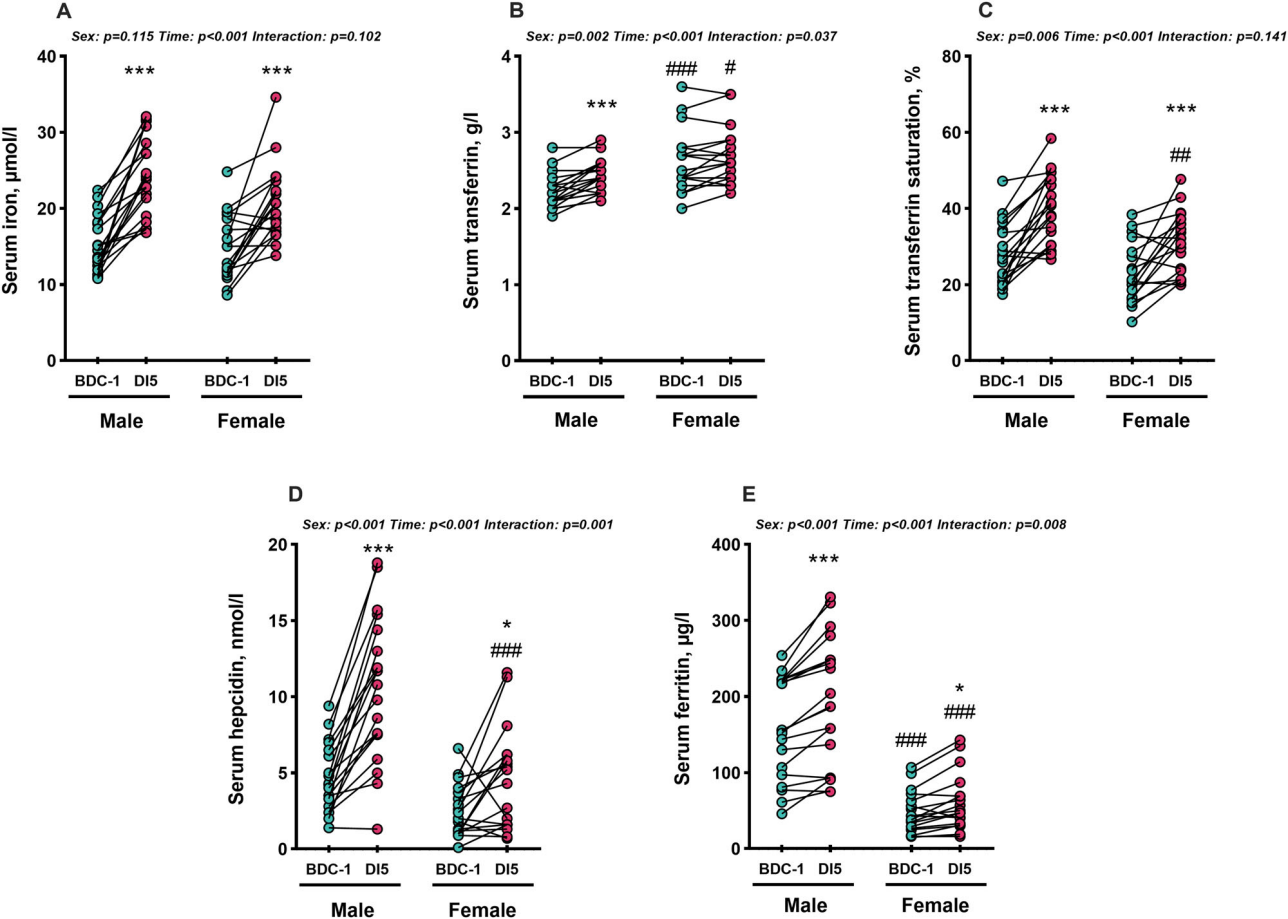
Effect of 5-day dry immersion on iron metabolism parameters in male and female participants. **A** Serum iron, **B** Serum transferrin, **C** Serum transferrin saturation, **D** Serum hepcidin, **E** Serum ferritin. Data are represented as individual values 1 day before dry immersion (BDC-1) and on the 5th day of DI (DI5). Data were analyzed with two-factor ANOVA for repeated measures followed by Tukey’s post hoc test in the case of global significance. Significant differences compared to BDC-1: *p < 0.05; **p < 0.01, ***p < 0.001. Significant differences between male and female participants in each time point: ^#^*p* < 0.05, ^##^*p* < 0.01, ^###^*p* < 0.001. BDC baseline data collection, DI dry immersion.

In the basal condition, before exposure to dry immersion (i.e., BDC-1), serum iron and transferrin saturation levels do not differ between the sexes (Fig. 1A, B). However, female volunteers exhibit lower serum ferritin levels (*p* < 0.001; Fig. 1E) and higher serum transferrin levels compared to male counterparts (*p* < 0.001; Fig. 1B), confirming sex-specific differences that are generally associated with higher iron stores in male participants^12^. Taken together, these findings suggest that, in the basal state, female participants: (1) have the same iron availability levels in plasma as males, and (2) unlike males, exhibit lower body iron stores. In the absence of an active iron excretion mechanism in humans, this could be attributed to menstrual bleeding, which constitutes an important source of iron release in premenopausal females^15^.

On the 5th day of dry immersion, both sexes exhibited increased serum iron levels ( + 57% for male and +40% for female participants, Time: *p* < 0.001, Fig. 1A), as well as an increase of serum transferrin levels, which is more pronounced in male compared to female participants ( + 8.5% vs. 3.2%, compared to basal situation, Time: *p* < 0.001, interaction: *p* = 0.037, Fig. 1B). Concomitantly, in both male and female participants, the rise in transferrin saturation levels after dry immersion ( + 12.2 and +8.0%, Time: *p* < 0.001, Fig. 1C) clearly indicates an increased plasma iron availability. These data support that simulated microgravity promotes an increase of plasma iron availability during the first days of exposure in both sexes, confirming our data previously collected in men and more recently in a smaller group of women^5,13^. Spaceflight and simulated microgravity are well-known to induce anemia, characterized by a decrease in hemoglobin mass in both sexes^13,16–18^. In the present study, we observed a significant decrease in hemoglobin mass in female participants after 5 days of simulated microgravity (−8.0%, Table 1), but a twofold lesser decrease in male participants (−3.9%, Table 1). The decrease in hemoglobin mass has previously been described as possibly being affected by repeated venipuncture. In our study, the amount of blood samples was rigorously conducted in an identical manner for each subject and cannot explain this difference between the sexes. Given that 65–70% of total body iron is bound within hemoglobin of red blood cells, this earlier reduction of total hemoglobin mass in female subjects, already observed during bed rest experiments^13^, suggests that men and women may experience different magnitude of iron metabolism alteration or misdistribution during the first days of simulated microgravity. In addition, with 20–25% of total body iron distributed in muscle myoglobin, muscle atrophy, which occurs earlier in female compared to male participants exposed to simulated microgravity^13,19–21^, could also contribute to the sex differences in the extent of plasma iron availability alterations.

**Table 1:** Effects of 5 days of dry immersion on blood parameters, serum hsCRP, and sex hormone levels in male and female participants^a^

	BDC-1		DI5		Two-factor RM ANOVA^b^		
	Male subjects	Female subjects	Male subjects	Female subjects	Sex	Time	Sex*Time
RBC, 10^6^ per pL	4.96 ± 0.35	4.30 ± 0.21^###^	5.69 ± 0.30***	4.81 ± 0.27^###^,***	*p* < 0.001	*p* < 0.001	*p* = 0.008
Reticulocyte count, %	0.90 ± 0.19	1.15 ± 0.47	0.98 ± 0.30	1.24 ± 0.46	*p* = 0.031	NS	NS
MCV, fl	91 ± 3	92 ± 4	91 ± 3	92 ± 3	NS	NS	NS
MCH, pg	31.4 ± 1.1	30.8 ± 1.1^###^	31.4 ± 1.1	30.9 ± 1.0^###^	NS	NS	NS
MCHC, g per dL	34.6 ± 0.4	33.6 ± 0.4	34.6 ± 0.3	33.6 ± 0.6	*p* < 0.001	NS	NS
Total Hb mass, g	817 ± 94	598 ± 112^###^	783 ± 96	542 ± 74^###^	*p* < 0.001	*p* = 0.001	NS
Δ Total Hb mass, %	-	-	−3.9 ± 7.6	−8.0 ± 12.1	NS	*p* = 0.001	NS
Relative Hb mass, g per kg	11.39 ± 1.43	10.19 ± 1.77^#^	11.13 ± 1.38	9.48 ± 1.25 */^##^	*p* = 0.004	*p* = 0.015	NS
Δ Relative Hb mass, %	-	-	−1.98 ± 7.77	−5.71 ± 12.6*	NS	*p* = 0.035	NS
Plasma volume, mL	3339 ± 457	3209 ± 671	2631 ± 457***	2402 ± 437***	*p* = 0.067	*p* < 0.001	NS
hs CRP, mg per L	0.26 ± 0.21	0.51 ± 0.58	0.54 ± 0.39***	0.56 ± 0.50	NS	*p* < 0.01	0.016
Total testosterone, nmol per L	20.85 ± 6.87	0.88 ± 0.27^###^	21.72 ± 6.70	1.02 ± 0.32^###^	*p* < 0.001	NS	NS
Oestradiol, pg per mL	-	91.59 ± 59.34	-	101.97 ± 56.91	-	-	-

*BDC* baseline data collection, *DI* dry immersion, *RBC* red blood cells, *MCV* mean corpuscular volume, *MCH* mean corpuscular hemoglobin, *MCHC* mean corpuscular hemoglobin content, *RM* repeated measure, *Hb mass* hemoglobin mass, *hsCRP* high sensitive C-reactive protein.

^a^Values are mean +/− SD for both sexes. Male and female participants compared with respective baseline: *p < 0.05; **p < 0.01, ***p < 0.001. Male compared to female participants in each time point: ^#^*p* < 0.05, ^##^*p* < 0.01, ^###^*p* < 0.001. *N* = 17 male and 18 female participants for CO-rebreathing data (tHb, relative Hb, Plasma volume); *n* = 19 male and 18 female participants for all other variables.

^b^Data were analyzed with two-factor RM ANOVA. For all significant global effects, Tukey’s post hoc test was used to detect differences between baseline and DI5, and between sexes.

**Fig. 2:**
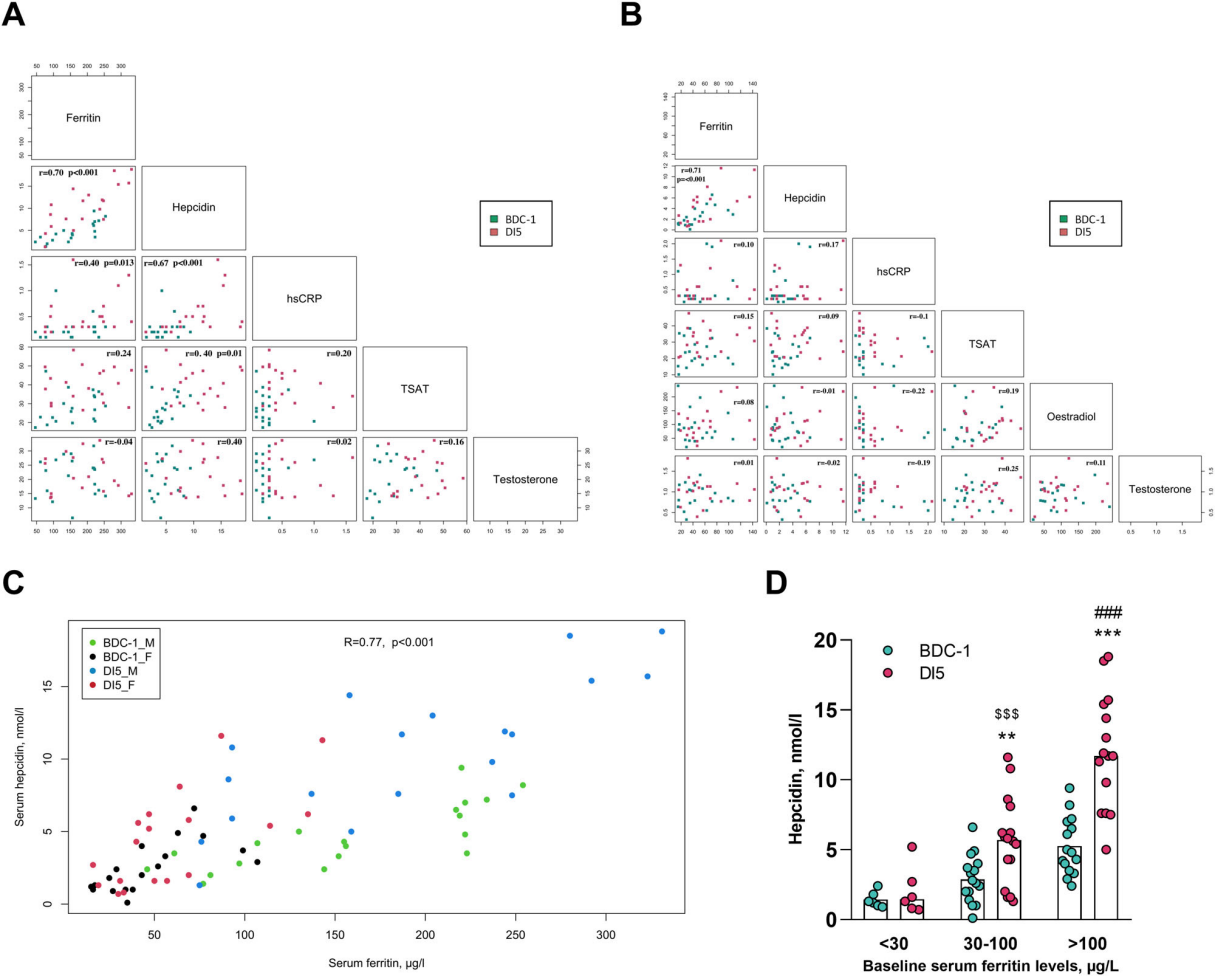
Relationship between serum hepcidin levels, iron metabolism, and hormonal status. **A** correlation matrix of serum hepcidin levels correlated with iron metabolism parameters, serum hsCRP, and sex hormones levels from male and **B** female participants before and at the end of 5-day dry immersion. **C** scatter plot between serum hepcidin and serum ferritin levels from male and female participants before and at the end of dry immersion, **D** serum hepcidin levels (male and female participants) before and at the end of DI with baseline serum ferritin levels <30 μg per L ( <30), between 30–100 μg per L (30–100), and >100 μg per L ( >100). Data are represented as individual values 1 day before dry immersion (BDC-1) and on the 5th day (DI5). Data were analyzed with 2-factor ANOVA for repeated measures followed by Tukey’s post hoc test in the case of global significance. Significant differences compared to BDC-1: **p < 0.01, ***p < 0.001. Significant differences compared to serum ferritin <30 per g per L: ^$$$^*p* < 0.001. Significant differences compared to serum ferritin between 30 and 100 µg per L: ^###^*p* < 0.001.

Alongside the increase in plasma iron availability, both sexes exhibit an increase in serum ferritin levels (Time: *p* < 0.001 compared to baseline, Fig. 1E), with however a greater increase observed in male compared to female participants after 5 days of dry immersion ( + 33.9 ± 27.0 vs. +13.6 ± 15.2 µg per l, Interaction: *p* = 0.008, respectively). Whether this increase in ferritin levels is solely attributable to enhanced iron stores within cells remains to be elucidated. Indeed, while an increase of serum ferritin levels is also observed during inflammation^22^, it must be emphasized that serum hsCRP levels, which reflect acute inflammation, do not increase in female participants. In contrast, a slight but significant increase is observed in male participants, although it remains within normal ranges (Table 1). Moreover, in male subjects, serum hsCRP levels are weakly, but significantly, correlated with serum ferritin levels (*r* = 0.4, *p* = 0.013, Fig. 2A). These data suggest that during the initial days of exposure to simulated microgravity, the increase of serum ferritin levels in males could be partially related to mild subclinical inflammation, in addition to an increase of body iron-storage and/or cytolysis favoring ferritin release into the serum.

Hepcidin, mainly synthesized by the liver in response to increased hepatic iron stores and inflammation^10^, is well-known to decrease serum iron concentrations by limiting the activity of ferroportin, the cellular iron exporter. Our data show that, besides the rise of serum iron levels after 5 days of dry immersion, both sexes exhibit an increase in serum hepcidin levels (Time: *p* < 0.001, Fig. 1D), more pronounced in male than in female participants ( + 5.9 ± 3.5 vs. +2.0 ± 3.1 nmol per L, interaction: *p* = 0.001, Fig. 1D). Notably, this hepcidin response in female subjects is heterogeneous, with a “responder” group (i.e., increase in serum hepcidin levels) and a “non-responder” group (i.e., no increase in serum hepcidin levels), whereas male participants consistently show an increase of serum hepcidin levels. These observations raise questions about (1) the source(s) of iron released into the plasma, given that a decrease in hepcidin expression is ruled out, and (2) the mechanisms behind the increase of serum hepcidin levels. Regarding the source of serum iron increase, as previously discussed, muscle atrophy and/or modification of blood parameters could at least contribute partly to this rise. Regarding the mechanisms of hepcidin increase, it is well reported that similarly to CRP, hepcidin expression is induced during an inflammatory state via the IL-6/STAT3 pathway^23^. Even though hsCRP levels only slightly increase in male subjects during dry immersion, the strong correlation between hepcidin and hsCRP values (*r* = 0.67, *p* < 0.001, Fig. 2A) suggests that an inflammatory state could elevate serum hepcidin levels during exposure to microgravity. Concurrently, a strong correlation is observed between serum hepcidin and ferritin levels in both sexes (*r* = 0.70, *p* < 0.001, *r* = 0.71, *p* < 0.001, respectively, Fig. 2A, B), with ferritin levels also known to be induced by inflammation. Importantly, we observed that participants with baseline serum ferritin values below 30 µg per L (all female participants) do not show an increase in serum hepcidin levels (i.e., the non-responders), whereas the increase becomes more significant when serum ferritin values at baseline exceed 100 µg per L (Fig. 2D). This could suggest that iron stores were lower in these females before exposure to dry immersion, thus limiting the impact of a sub-inflammatory state on hepcidin levels. It is important to point out that no significant correlation was identified between the menstrual cycle and the “responder”/“non-responder” groups. Moreover, despite the fact that hepcidin synthesis has been reported to be downregulated by estrogen levels^24^, no statistically significant relationship is found between serum estrogen levels and serum hepcidin levels in female subjects (*r* = −0.01; *p* = 0.53; Fig. 2A). Furthermore, menstrual losses, a potential source of iron loss in most premenopausal females^25^, could not be a key factor of the modulation of iron parameters during the 5 days of dry immersion, as no female participants experienced menstrual bleeding (detailed in Robin et al.^26^). Altogether, our findings suggest that the modulation of iron metabolism parameters is related to intricate factors and that unidentified factors could also contribute to the sex differences in the magnitude of iron metabolism responses to simulated microgravity.

In summary, our data indicate that short-term exposure to simulated microgravity results in increased iron availability, serum ferritin, and hepcidin levels in both sexes, pointing to an alteration of iron metabolism. It is crucial to uncover the origin of this iron misdistribution and to investigate whether iron could accumulate in the organs of male and female astronauts. These findings highlight the need to investigate the longer-term effects of microgravity, combined with exposure to cosmic radiation, on iron redistribution in both men and women. A thorough understanding of these mechanisms in both male and female astronauts will facilitate the development of dietary and physical activity strategies as well as menstrual management protocols aboard the ISS and Gateway, aiming to prevent excessive accumulation of body iron.

## Methods

### Study design

The study was sponsored by the European Space Agency in collaboration with the Centre National d’Etudes Spatiales. The VIVALDI study was approved by the national Comité de Protection des personnes d’Ile de France II (ID-RCB: 2021-A00705-36) and registered on ClinicalTrials.gov under NCT05043974. The VIVALDI2 study received approval from the national Comité de Protection des personnes d’Ile de France VII (ID-RCB: 2022-A00881-42) and was registered on ClinicalTrials.gov under NCT05493176. These controlled clinical trials were conducted at the MEDES space clinic in France between September 2021 and November 2022 and involved the recruitment of 20 young healthy female participants for VIVALDI and 20 young healthy male participants for VIVALDI2. All participants were informed about the experimental procedures and provided written consent. One female participant left the protocol on the first day of immersion due to a technical issue (a technical problem with the bath lifting platform which could not be fixed quickly without emptying the bath leading to the decision to stop the study for this participant), and another could not be included for regulatory reasons (this participant unexpectedly was still during the exclusion period following participation in a previous unrelated clinical trial). One male participant withdrew from the study on the third day of immersion due to severe back pain. They were excluded from the analysis. Consequently, the data analysis finally included 18 healthy female and 19 healthy male participants (Supplemental Table 1). Detailed inclusion and exclusion criteria can be found in the Supplementary Information. Each clinical study included 4 days of baseline data collection (BDC-4 to BDC-1), 5 days of dry immersion (DI1 to DI5), and 2 days of ambulatory recovery (R + 0 and R + 1). The dry immersion protocol is described in previous studies^5,26^. Antecubital venous blood samples were collected in the morning before breakfast one day before (BDC-1) and on the 5th day of dry immersion (DI5). In each campaign (Vivaldi 1&2), 340 mL of blood was collected per subject during the study. This included 111 mL before (BDC-4 to BDC-1), 171 mL during (DI1 to DI5), and 58 mL after dry immersion (R + 0 to R + 1). Plasma and serum samples were analyzed for blood count, iron metabolism parameters, and hormonal status.

### Iron metabolism parameters

Iron metabolism parameters were measured in samples collected at BDC-1 and DI5 at the biochemistry laboratory (Rennes University Hospital, France). Serum iron concentrations were measured using standard colorimetric methods, serum transferrin concentrations by immunoturbidimetry, and serum ferritin concentrations by electro-chemiluminescence immunoassay. The total (or transferrin) iron binding capacity (TIBC; in µmol per L) was calculated (transferrin in g per L x 25) to determine transferrin saturation (%) as (iron in µmol per L/TIBC) x 100.

### RBC parameters

RBC and reticulocyte counts, mean corpuscular hemoglobin (MCH), mean corpuscular volume (MCV), and mean corpuscular hemoglobin concentration (MCHC) were measured in blood samples collected at BDC-1 and DI5 by the Advia 2120 (Siemens Healthcare Diagnostics, Deerfield, Illinois, USA), an automated hematology analyzer. Total hemoglobin (tHb) mass and plasma volume were measured using the optimized carbon monoxide (CO) rebreathing method (oCORM) at DI1 before the onset of immersion and at DI5. After an initial resting period in the supine position, a baseline 2.6-mL EDTA blood sample was taken from an antecubital vein (S-Monovette®, Sarstaedt AG & Co.) and the carboxyhemoglobin percentage (%HbCO) was analyzed immediately using a hemoximeter (ABL 825, Radiometer). The blood sample was transferred to a capillary tube and centrifuged at 3000 × *g* for 10 min at room temperature, and the ratio was measured and expressed as a decimal or percentage fraction. Participants initially breathed 100% oxygen for 4 min to flush the nitrogen from the airways, followed by administration of a bolus (1.5 mL per kg for male and 1.0 mL per kg for female participants) of 99.997% chemically pure CO (CO N47, Air Liquide). Participants rebreathed this gas mixture for 10 min. Then, another 2.6-mL blood sample was collected to measure again the %HbCO. The change between %HbCO values was used to calculate the tHb mass, by taking into account the CO amount that remained in the rebreathing circuit. For 2 male subjects, oCORM data were excluded from analysis (aberrant values); tHb and plasma volume data are shown for 17 male and 18 female participants.

### Hormones and cytokines

Serum hepcidin was measured using the Hepcidin-25 (human)—Lyophilized Antiserum for EIA/ELISA Kit (catalog number: S-1337, Peninsula Laboratories International, Inc.) according to the manufacturer’s instructions. Serum total testosterone levels were measured by chemiluminescence using the Siemens ADVIA Centaur Testosterone II (TSTII) assay with the ADVIA Centaur® CP analyzer (Siemens Healthcare Diagnostics Inc.). Serum oestradiol concentrations were determined using a radioimmunoassay kit according to the instructions of the manufacturer (Immunotech, Beckman Coulter). Serum concentrations of high-sensitivity C-reactive protein (hsCRP) were measured using latex-enhanced immunoturbidimetric (Advia Chemistry XPT systems, Siemens).

### Statistical analyses

Graphical data are represented as individual values and table data are represented as mean ± SD. To determine the effect of dry immersion and sex, each experimental group (male and female participants) was compared using two-factor ANOVA (i.e., general linear model) for repeated measures with the Greenhouse-Geisser correction followed by the Tukey multiple comparison test with the adjusted *p*-value. Relationships between variables of interest were examined using the Pearson correlation coefficient for normal distribution or Spearman for non-normal distribution. Data were analyzed using GraphPad Prism version 8.20 (GraphPad Software).

### Supplementary information

Supplementary information

## Data Availability

The data supporting the conclusions of this article are available from the corresponding author upon reasonable request.
