# Supplementary material for: Dry immersion rapidly disturbs iron metabolism in men and women: results from the VIVALDI studies
Source: NPJ Microgravity. 2024 Jun 15;10:68. doi: 10.1038/s41526-024-00399-z (PMC11180090; doi:10.1038/s41526-024-00399-z)
Supplement: Supplementary file 1 — Supplementary information [file 41526_2024_399_MOESM1_ESM.pdf]

Dry immersion rapidly disturbs iron metabolism in men and women: results from the VIVALDI studies- Horeau et al.

Online Supplementary Material

Supplemental Table 1. Baseline characteristics at BDC-1 in male and female participants[1]

	**Males**	**Females**
	**n=19**	**n=18**
**Age (years)**	28 ± 4.32	28.78 ± 4.70
**Height (cm)**	176.63 ± 4.21	164.75 ± 5.83
**Weight (kg)**	71.96 ± 6.7	59.32 ± 6.25
**BMI (kg/m^2)**	23.05 ± 1.88	21.84 ± 1.77

[1] Values are presented as mean ± SD for both sexes, (n=19 males, n=18 females).

MATERIAL AND METHODS

Inclusion Criteria:

- Healthy female and male volunteers (see below the description of medical tests and laboratory analysis performed at the selection visit),
- Age 20 to 40,
- No overweight nor excessive thinness with BMI (weight Kg/ height m2) between 20 and 26,
- Height between 158cm and 185 cm,
- For females only : regular menstrual cycles and cycles lasting between 20 and 35 days
- For females only: without oestroprogestative contraception (i.e., oral progestative contraception, IUDs, implants or absence of contraception are allowed),
- Certified as healthy by a comprehensive clinical assessment (detailed medical history and complete physical examination): in particular, free from any chronic disease or any acute infectious disease or cardiovascular, neurological, ENT (especially orthostatic hypotension and vestibular disorders), orthopaedic or musculoskeletal disorders,
- Fitness level assessment: 35 ml/min./kg $<$ VO2max $<$ 55 ml/min./kg,
- Non active smokers,
- No alcohol, or drug addiction, and no medical treatment (with the exception of the aforementioned accepted means of contraception),
- Covered by a Health Insurance System,
- Having signed the informed consent,
- Free from any engagement during the study.

Exclusion Criteria:

- Any history or presence of clinically relevant cardiovascular, neurological or ENT (especially orthostatic hypotension and vestibular disorders), any chronic disease; any acute infectious disease, in particular,
- Symptomatic orthostatic hypotension whatever the decrease in blood pressure,
- Asymptomatic postural hypotension defined by a decrease in SBP equal to or greater than 20 mmHg within 3 minutes when changing from the supine to the standing position,
- Cardiac rhythm disorders,
- Hypertension,

- Chronic back pains,
- Vertebral fracture, scoliosis or herniated disc,
- Glaucoma,
- Self-reported hearing problems,
- History of migraines,
- History of hiatus hernia or gastro-esophageal reflux,
- History of thyroid dysfunction, renal stones, diabetes,
- History of head trauma,
- Abnormal result for lower limbs echo-doppler,
- History of genetic muscle and bone diseases of any kind,
- Past records of thrombophlebitis, family history of thrombosis or positive response in thrombosis screening procedure (anti thrombin III, S-protein, C-protein, factor V Leiden mutation and the mutation 20210 of the prothrombin gene),
- Women who have stopped breastfeeding within 2 months before the start of the study,
- Women who have undergone an abortion within 3 months before the start of the study,
- Women who have irregular menstrual cycles or cycles lasting less than 20 days or more than 35 days,
- Women who use oestroprogestative contraception,
- Women who recently implemented or changed in hormonal contraceptive (in the last 6 months),
- Bone mineral density: T-score $\leq$ -1.5,
- Poor tolerance to blood sampling,
- Having given whole blood (more than 8ml/kg) in a period of 8 weeks or less before the start of the experiment, or having given whole blood more than 3 times in the past year,
- Significant history of allergy, especially no dermatological allergy,
- History of food allergy,
- Significant anomaly detected in the biological analysis,
- Positive reaction to any of the following tests: HVA IgM (hepatitis A), HBs antigen (hepatitis B), anti-HVC antibodies (hepatitis C), anti-HIV1+2 antibodies,
- Vegetarian or vegan,
- Refusal to give permission to contact her general practitioner,

- Subject who, in the judgment of the investigator, is likely to be non-compliant during the study, or unable to cooperate because of a language problem or poor mental development,
- Subject already participating or in the exclusion period of a clinical research,
- Subject who has received more than 4500 Euros within 12 months for being a research subject,
- Subject who cannot be contacted in case of emergency,
- MRI contraindications History or active claustrophobia, Osteosynthesis material, presence of metallic implants or any other contra-indication for MRI, Allergy to Gadolinium.
- Pregnant women (urine pregnancy test performed at the selection visit and on the day of arrival in the facility),
- Women during childbirth and breastfeeding mothers,
- Persons deprived of their liberty by an administrative or judicial decision,
- Persons under involuntary psychiatric care,
